# Mammogram Uptake from Social Determinants of Health Can Be Lost in Translation to Individual Patients

**DOI:** 10.21203/rs.3.rs-3298459/v1

**Published:** 2023-08-31

**Authors:** Matthew Davis, Kit Simpson, Vanessa Diaz, Alexander V. Alekseyenko

**Affiliations:** Medical University of South Carolina

## Abstract

**Purpose:**

The objective of this study is to describe patterns in barriers to breast cancer screening uptake with the end goal of improving screening adherence and decreasing the burden of mortality due to breast cancer. This study looks at social determinants of health and their association to screening and mortality. It also investigates the extent that models trained on county data are generalizable to individuals.

**Methods:**

County level screening uptake and age adjusted mortality due to breast cancer are combined with the Centers for Disease Controls Social Vulnerability Index (SVI) to train a model predicting screening uptake rates. Patterns learned are then applied to de-identified electronic medical records from individual patients to make predictions on mammogram screening follow through.

**Results:**

Accurate predictions can be made about a county’s breast cancer screening uptake with the SVI. However, the association between increased screening, and decreased age adjusted mortality, doesn’t hold in areas with a high proportion of minority residents. It is also shown that patterns learned from county SVI data have little discriminative power at the patient level.

**Conclusion:**

This study demonstrates that social determinants in the SVI can explain much of the variance in county breast cancer screening rates. However, these same patterns fail to discriminate which patients will have timely follow through of a mammogram screening test. This study also concludes that the core association between increased screening and decreased age adjusted mortality does not hold in high proportion minority areas.

## Objective

The objective of this study is to describe patterns in social determinants of health and their association with female breast cancer screening uptake, age adjusted breast cancer mortality rate and the extent that models trained on county data are generalizable to individuals.

## Introduction

Cancer is the second leading cause of death in United States. According to the CDC, female breast cancer in 2019 was the second leading cause of death at 19.4/100,000 with 129.7/100,000 new cases per year^1^. Improving breast cancer screening uptake is a key strategy to reducing mortality by enabling early detection and intervention^1^. This study uses machine learning to detect and quantify patterns in the relationships between SODH, mammogram screening and follow-up, and age adjusted breast cancer mortality rates. We test the hypothesis that models developed using county-level SODH data improve screening predictions for individual patients. It is well documented that SODH measures are valid for identifying population health issues, however, the value of these measures for individual risk prediction useful for identifying individuals in need of special outreach and resources to complete screening has not been well described.

### Background

The relationship between population level SODH and age adjusted breast cancer mortality have been well documented in literature. This was summarized by a systematic review published by Gerend et al. in 2008 that identified poverty, social justice and social factors as contributors to screening uptake differences between African American and White/Caucasian patients ^2^. However, sources of information indicating SDOH risk can include individual billed ICD10-CM codes, self-reported data or geographically attributed population-level data. Our research study will extend findings published in 2018 by Heller et al^3^ that showed variation in the ability of County Health Rankings data to identify the percentage of female Medicare enrollees 67–69 years old per county who had at least one mammogram and specifically lower screening uptake rates in counties associated with higher poverty rates. The 2018 study also reported that screening uptake was positively correlated to the proportion of Medicare patients in a particular county with some college education^3^. Heller et al also showed that college education was negatively correlated with the age adjusted mortality per county^3^. Our study extends this work by utilizing additional data sources for counties (the CDC CVI), applying new statistical methods to address intercorrelations between variables, and tests the hypothesis that patterns associated with county screening uptake data can be used to discriminate between patients that will and will not have timely follow through on mammogram screening results. We include the CDC SVI which incorporates 15 social factors, including unemployment, minority status, and disability that are calculated by census tract and county. These data provide robust measures representing social risk factors. Tree Augmented Naive Bayesian networks are used to reduce the SVI to essential features associated with screening uptake and the age adjusted breast cancer mortality outcome. Testing these models not only on population-level data, but for individual patient prediction, will provide insights into the generalizability of the patterns learned.

### Methods

This project was reviewed and approved by MUSC IRB (Protocol number Pro00101494).

**Figure 1:**
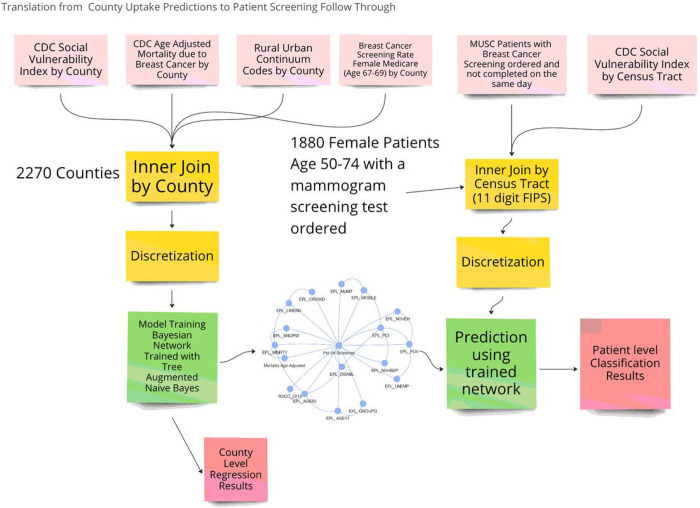
Study design for translation county level mammogram screening uptake to patient level screening follow through. The top boxes show 4 county level data sets being merged and discretized, and used to tran a model predicting county breast cancer screening uptake. The remained CDC SVI Census tract data set was then merged with MUSC Patient data, where the trained model was used to make predictions on which patients would actually follow through on mammogram screening.

Data for this study used four publicly available county level data sets to train a Tree Augmented Naive Bayes (TAN) network, and a fifth dataset of individual patient data to learn screening uptake patterns and make predictions about a county’s breast cancer screening uptake, and age adjusted mortality. This study also tested the strength of these learned pattern’s ability to discriminate between patients that followed through with having a breast cancer screening test. The usage of the datasets, and the transfer of the model trained on county data to individual patients is shown in Figure 1.

The first four data sets were used for initial model training include (1) the County level 2018 CDC/ATSDR Social Vulnerability Index (SVI)^4^, (2) the County Health Rankings data to identify the percentage of Medicare enrollees 67–69 years old per county who had at least one mammogram in 2015 sourced from the Dartmouth Atlas of health care^5^, (3) CDC WONDER Female Breast Cancer Mortality Rate, averaged 2010–2020 by county^6^ and (4) the United States Department of Agriculture Rural Continuum Codes (RUCC). The datasets were joined together using Federal Information Processing Standard County Code (FIPS). This combined data was discretized in the following way. Mammogram screening rates and age adjusted mortality due to breast cancer were binned in buckets with uniform width of 5%. CDC SVI Estimated Percentile (EPL) columns were rounded to the nearest 10 to create buckets with uniform width of 10%. Counties without age adjusted mortality, or screening rates not measured between 0 and 100% were masked from the data.

Bayesian networks are graph models that encode probabilistic uncertainty between nodes and have been used to represent relationships between variables and outcomes^7^. They consist of a directed acyclic graph (DAG) and a table of conditional probabilities between the nodes.

Bayesian networks require the assumption that all features are independent. given the class of the observation, which in this case would be the county’s mammogram screening uptake rate. It is doubtful that features in the CDC SVI such as poverty, income, education, and vehicle ownership percentiles per county would be conditionally independent with respect to cancer screening rates. To address this, the TAN model is used to force all features to be dependent on the screening uptake, and only one other feature. TAN is a network structure learning algorithm that relaxes the independence requirement and imposes a tree structure where all nodes initially share an edge with the class node and contains the variables interaction with other variables, limiting them to two parents^8^. This method greatly reduces the computation complexity required to learn the network. Both Bayesian networks and TANs require underlying data to be discrete in order to learn underlying network structure. A subset set of a graph known as the Markov blankets or Markov boundary around a particular node is defined as the subset of parents, children and parents of children of a particular node ^9^. The Markov blanket is thought of as the minimal set of information about a node, however, it is not unique ^10^.

The Bayesian network structure was learned using the BN Learn package with the TAN method, trained on the county level discretized data^11^. The network structure was then used to learn conditional probabilities between nodes and used independence testing to prune features with p-values greater than 0.2. The final network represented county associations between age adjusted mortality due to breast cancer, mammogram screening and the CDC SVI. The Markov blanket of the screening variable was used to subset the network. The results were analyzed with the linear weighted kappa score to quantify the extent the network learning produced predictions that agreed with the discretized screening at mortality variables. The individual level validation cohort was derived from electronic medical record data of Medical University of South Carolina (MUSC), and included females aged 50–74 at the time of at least one billed visit during the 2016–2019 time period with at least one breast cancer screening test ordered. The dataset had a target task of predicting which patients would follow through and complete the breast cancer screening test within 180 days. Patients completing the mammogram on the initial date that it was order were masked. The dataset consisted of 1880 female patients that had at least one mammogram screening test ordered with features describing comorbidity, demographic, self-reported personal and family cancer history as well as geographically linked (at the census track level) social variability derived from the 2018 CDC/ATSDR Social Vulnerability Index^4^.

The patient level data from MUSC was discretized using the same method as the data for the county level model. SVI tract level features were used to predict the screening uptake rate, and age adjusted mortality due to breast cancer. The predictions were compared to PCT or ICD10CM codes that indicated the patient had completed a mammogram screening during the study time frame. Performance metrics were calculated on the overall cohort of patients, and area under the curve of the receiver operating characteristic (AUC) was used as a primary metric to describe the model’s ability to discriminate between patients that completed screening vs. not after being ordered by a provider at different thresholds. An investigation was also conducted into how percentile percentage of minorities of a particular county an effect on the relationship between age adjusted mortality and screening. Mixed effects linear models were used with age adjusted mortality as a dependent variable, percent unscreened as an independent variable, and a flag indicating the county was at or above the 90^th^ percentile of the percentage minority having a random slope and intercept.

## Results

The county level data (joining the SVI, screening uptake data and breast cancer mortality data) resulted in a dataset containing 2,270 counties in the United States with 13 features and two outcomes. Summary statistics of the data are shown in Table 1. The result of joining the individual MUSC patient data to tract level SVI area shown in with summary statistics in Table 2.

We evaluated the individual-level variables for association with mammogram completion within 180 days. Counts, proportions of and fishers exact test results to show difference in proportion for each feature being true for case and controls are summarized in Table 3. Depression, anxiety and menopause/premenopausal billed ICD10-CM diagnoses codes all had strong associations with increased odds of failing to follow through on screening. Medicaid insurance also had an association with lower screening completion rates.

**Figure 2:**
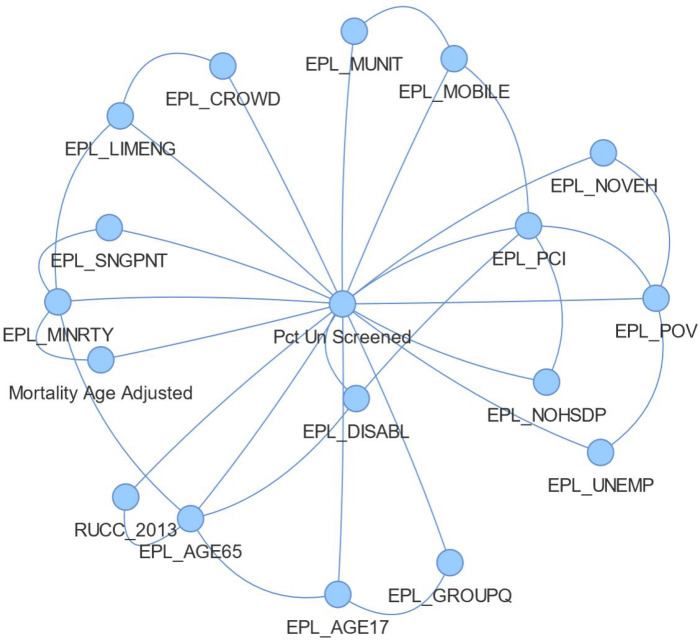
Network of Factors Influencing Mammogram Screening and Age Adjusted Mortality from Breast Cancer. The proportion of patients that went unscreened in a county the class node, which all other nodes have edges, due the TAN network constraints. This shows that percentile minority is a confounding the association of age adjusted mortality and proportion of patients in a county that have received mammogram screening. Also notable is that network structure learned encoded poverty, percentile income, percentile of not having high school diplomas, unemployment, and low vehicle ownership as being associated with each other.

**Figure 3:**
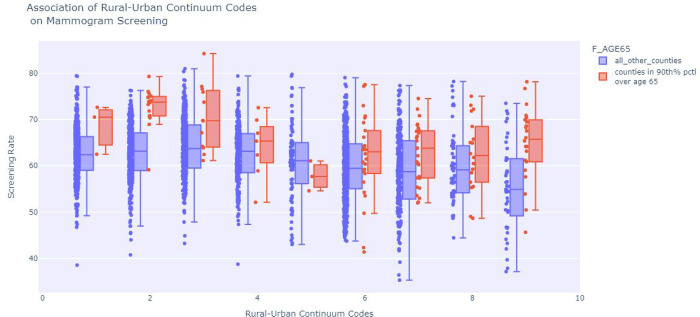
This shows the difference in mammogram screening uptake between counties with high proportion of people over age 65 vs not, for each RUC code with the 25th, 50th, and 75^th^ percentiles marked as the box, and the whisker bars as 1.5x the interquartile range. This demonstrates consistently higher screening rates in counties with older populations at each Rural-Urban Continuum level.

The network structure trained on the county data resulted in a host of associations between SVI features and screening shown in Fig. 2. This shows the associations learned between the percent of patients unscreened, age adjusted mortality and the CDC SVI. Each edge in the graph contains conventional probabilities between edges. The EPL_POV node, representing percentiles of persons in poverty is shown to have associations with lower vehicle ownership (EPL_NOVEH), lower income (EPL_PCI) higher unemployment (EPL_UNEMP) and lower mammogram screening uptake (Pct Un Screened). Also, shown in Fig. 2 is the strongest association to age adjusted mortality are mammogram screening uptake and percentile minority (EPL_MNTRY). The network learning algorithm found associations related to age adjusted mortality and screening uptake was confounded by the estimated percentile of minorities in a county. The network also revealed that estimated percentile of age over 65 was a confounding factor in the association between rural-urban continuum code and the proportion of female Medicare patients aged 67–69 without a mammogram screening in the prior two years. The positive correlation between the rural-urban continuum code had a Pearson coefficient 0.21 with p-value < 0.000, however, counties in the 90th percentile of age over 65% consistently had a lower percentage of unscreened individual as shown in Fig. 3, at almost all values of Rural-Urban continuum levels shown tabulated in Table 4.

Next, we used the network-derived features for screening outcome prediction. The resulting model had the following 16 feature inputs: EPL_POV, EPL_UNEMP, EPL_PCI, EPL_NOHSDP, EPL_AGE65, EPL_AGE17, EPL_DISABL, EPL_SNGPNT, EPL_MINRTY, EPL_LIMENG, EPL_MUNIT, EPL_MOBILE, EPL_CROWD, EPL_NOVEH, EPL_GROUPQ, RUCC_2013 of which descriptions are located in Table 3. The task of predicting the proportion of patients that went unscreened resulted in a weighted kappa of 0.82 and accuracy 0.79 predicting the proportion persons that went unscreened. This demonstrated a high level of agreement between the model’s predictions and the actual proportion of unscreened patients. The task of predicting age adjusted mortality due to breast cancer from the same network resulted in weighted kappa of 0.14 and accuracy of 0.57. This demonstrated relatively poor agreement between model predictions and age adjusted mortality, however since it was not the primary class node, the TAN network architecture limited the number of parent variables to two; percentile of minorities in a county (EPL_MINRTY) and proportion of unscreened patients. EPL_MINRTY was shown to be a confounding factor between screening uptake and age adjusted mortality due to breast cancer, additional regressions were conducted to quantify the associations. This experiment used mixed effects models with the county mammogram screening uptake rate as an independent variable, and the age adjusted mortality as the dependent, where random slope and intercepts were fit for counties flagged in of the 90th percentile of proportion minority.

For counties *not* in the 90th percentile of proportion minority, the resulting regression shows every 10% increase in a county’s screening rate, a 1.3 to 1.7 person per 100,000 decrease in age adjusted mortality would be expected with a p-value less than 0.001, and r-squared of 0.082. This shows a clear effect of decreasing age adjusted mortality when due to breast cancer, when screening is increased, for counties not in the highest percentiles of minorities.

**Figure 4:**
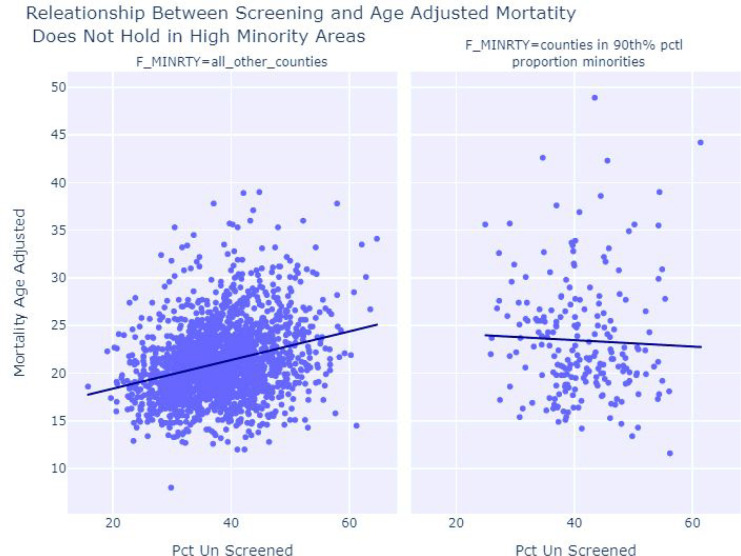
This shows female age adjusted mortality due to breast cancer plotted with the percent of persons that went unscreened. When the proportion of females that are un screened goes up, the age adjusted mortality also increases for areas not in the highest proportion on minority shown in the left panel. This association is no true for high proportion minority areas where the association of screening and age adjusted mortality is uncertain.

For counties *in* the 90th percentile of proportion of minorities, a 10% increase in a county’s screening rate would be associated with a −0.9 to 1.5 per person 100,000 change to the age adjusted mortality rate with p-value 0.58 and r-squared 0.001. This shows effect of increasing screening on age adjusted mortality is uncertain on the 209 counties, flagged as being the highest percentiles of minorities. Side by side regression results demonstrate the discrepancy in the screening and age adjusted mortality relationship in high proportion minority areas verses other areas are shown in Fig. 4. This breakdown of the relationship between screening and age adjusted mortality in high proportion minority areas suggests other unaccounted factors are influencing age adjusted mortality.

**Figure 5:**
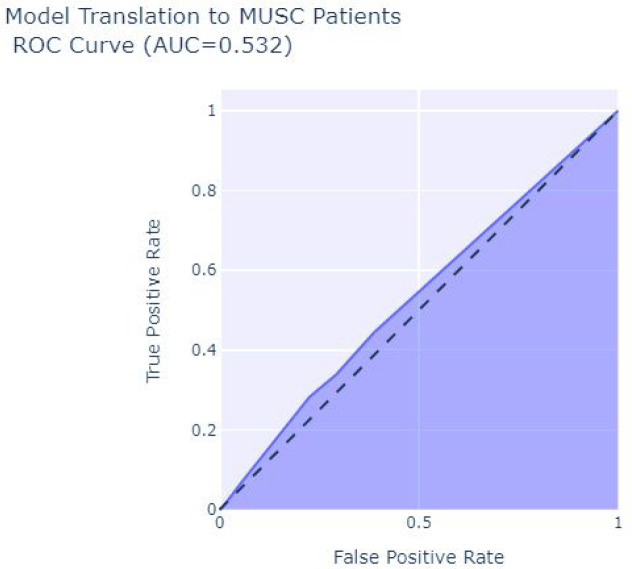
Model Trained on County Screening Uptake Have a Poor Ability to Discriminate Between Patients that Will or Will Not Complete a Mammogram Screening After Ordered by a Provided.

Results of translating the network model to patient with SVI data collected at the track level and using the predictions to rank the likelihood of patient completing a screening test after ordered within 180 days resulted in AUC score of 0.532 (0.524–0.54) and ROC shown in Fig. 5. This suggests that the network trained on county level data had little discriminative ability in predicting which patients would complete the screening test. The model preformed even worse specifically for the age 67–69 cohort (matching the age in county level CMS screening uptake metric) with an AUC of 0.42.

## Discussion

There were two key findings from this study. Firstly, county level social factors in the CDC SVI can predict patterns about county mammogram screening rates, however, they fail to make meaningful predictions about individuals. The county model’s fail to translate to individuals. Thus, models trained on county-level SDOH measures should not be assumed to provide useful insights about individual patient behavior. This finding is an example of the well-known concept of an ecological fallacy, where there is a mistaken assumption that statistical patterns derived from groups represent the individuals comprising those groups^12^. The associations were learned by the TAN network by county and applied to induvial patient screening follow-through with SVI attribution through census tract. This demonstrates this fallacy can arise during machine learning model development and application.

The second significant finding was that the association between screening uptake and age adjusted mortality measured in most counties, fails to hold in high minority density areas. The prediction breaks down in the cancer screening and age adjusted mortality relationship in high minority areas is profound. This means that it should not be assumed that increasing screening rates for high minority areas will have the same positive impact as that observed for areas with few minority residents. The implication of this is that one of the primary population health improvement tools used to alleviate the burden of breast cancer mortality does not appear to be adequate for high minority density areas.

Limitations of this study must be considered. Chief among them is the difference between county and individual screening measure definitions. The county level screening metric was measured for females aged 67–69 with at least one mammogram screening in the prior two years, whereas the individual patient measure assessed whether a patient completed a mammogram screening with 180 days of having one ordered by a provider. This choice was made to understand whether the model would be useful in clinical practice, where uptake in the short term would be more relevant.

The primary limitation to this work is that the predictions made about MUSC patients had CDC SVI attributes that were attributed to patients at the census tract level. This associates aggregated information from an entire census tract to an individual. This study provides some evidence in the mammogram screening rate, and individual predictions should not be made based on this level of information.

This study sourced patient screening follow-through from a single health system (MUSC) and it is unclear whether results would be applicable at other health systems.

The learning algorithm itself presented limitations. The CDC SVI is a robust measurement representing geography based social determinants of health, however the TAN structure learning algorithm limits the number of parents that a child of the class node can have to two, and only the strongest associations are returned. This leaves open the possibility that other associations between the SVI and age adjusted mortality are present but being pruned considering the strength between the association of percentile rank of proportion of minorities, screening uptake and age adjusted mortality.

Future studies are needed to investigate additional factors such as stage at diagnoses, aggressiveness of care, social stigma, and access to treatment that may be differentiating the screening and mortality relationship in high minority areas, vs other areas.

## Conclusion

This study shows the ability to use the CDC SVI to understand a significant portion of the variance in county level mammogram screening uptake. However, models trained on these data were shown to be ineffective at discriminating between which patients would complete a mammogram screening within six months after having one ordered from a health care provider. This suggests the need to use multiple data sources when developing breast cancer screening initiatives, as county level factors and individual level factors may supplement each other. This study also demonstrated that the core association between increased screening and decreased age adjusted mortality does not hold in high proportion minority areas. This suggests additional barriers not being captured by CDC SVI are contributing to the age adjusted mortality rate. In those areas, screening increases alone may be insufficient to decrease the burden of mortality due to breast cancer.

## Figures and Tables

**Table 1:** Summary statistics for county level mammogram screening uptake.

Statistics for 2270 Counties	Quantile 25	Quantile 50	Quantile 75	definition
**EPL_POV**	0.280	0.523	0.753	Percentile Percentage of persons below poverty estimate
**EPL_UNEMP**	0.319	0.542	0.756	Percentile Percentage of civilian (age 16+) unemployed estimate
**EPL_PCI**	0.248	0.497	0.740	Percentile per capita income estimate
**EPL_NOHSDP**	0.269	0.507	0.733	Percentile Percentage of persons with no high school diploma (age 25+) estimate
**EPL_AGE65**	0.223	0.427	0.659	Percentile percentage of persons aged 65 and older estimate
**EPL_AGE17**	0.270	0.508	0.746	Percentile percentage of persons aged 17 and younger estimate
**EPL_DISABL**	0.243	0.483	0.730	Percentile percentage of civilian noninstitutionalized population with a disability estimate
**EPL_SNGPNT**	0.327	0.553	0.774	Percentile percentage of single parent households with children under 18 estimate
**EPL_MINRTY**	0.292	0.527	0.750	Percentile percentage minority (all persons except white, non-Hispanic) estimate
**EPL_LIMENG**	0.300	0.537	0.760	Percentile percentage of persons (age 5+) who speak English “less than well” estimate
**EPL_MUNIT**	0.338	0.584	0.805	Percentile percentage housing in structures with 10 or more units estimate
**EPL_MOBILE**	0.226	0.473	0.734	Percentile percentage mobile homes estimate
**EPL_CROWD**	0.291	0.513	0.739	Percentile percentage households with more people than rooms estimate
**EPL_NOVEH**	0.294	0.534	0.766	Percentile percentage households with no vehicle available estimate
**EPL_GROUPQ**	0.267	0.517	0.758	Percentage of persons in group quarters estimate
**RUCC_2013**	2.000	4.000	6.000	USDA 2013 Rural Urban Continuum Code
**Pct Un Screened**	33.260	38.251	42.959	Percentage of Female Medicare enrollees 67–69 years old per county who did not have at least one mammogram in the prior two years 2015
**Mortality Age Adjusted**	18.700	20.800	23.300	Female Age Adjusted Mortality Rate from Breast Cancer per 100,000 averaged 2010–2020

**Table 2:** Summary Statistics SVI Statistics for MUSC Patients with Mammogram Screening Ordered

1880 MUSC Patients Age 50–74	mean	std	25%	75%
**EPL_POV**	0.422	0.297	0.173	0.637
**EPL_UNEMP**	0.336	0.278	0.124	0.545
**EPL_PCI**	0.354	0.261	0.162	0.576
**EPL_NOHSDP**	0.341	0.270	0.120	0.571
**EPL_AGE65**	0.542	0.240	0.363	0.726
**EPL_AGE17**	0.329	0.269	0.117	0.456
**EPL_DISABL**	0.391	0.239	0.219	0.543
**EPL_SNGPNT**	0.364	0.282	0.145	0.564
**EPL_MINRTY**	0.481	0.230	0.332	0.632
**EPL_LIMENG**	0.284	0.243	0.000	0.529
**EPL_MUNIT**	0.533	0.285	0.379	0.781
**EPL_MOBILE**	0.561	0.317	0.474	0.775
**EPL_CROWD**	0.306	0.238	0.000	0.466
**EPL_NOVEH**	0.395	0.272	0.172	0.562
**EPL_GROUPQ**	0.366	0.318	0.000	0.583
**RUCC_2013**	2.084	0.501	2.000	2.000
**RUCC_2013**	2.084	0.501	2.000	2.000
**Failed to Complete Screening**	0.409	0.492	0.000	1.000
**Mortality Age Adjusted by county**	19.382	1.273	19.100	19.100
**Age At Visit**	61.787	6.744	56.000	67.000
**Elixhauser Comorbidity Count**	0.373	0.957	0.000	0.000

**Table 3:** Differences in demographic and clinical characteristics of MUSC patients with a mammogram screening ordered.

	1880 MUSC Female Patients Age 50–74	True Among Cases^1^	True Amount Controls	Proportion Among Cases	Proportion Among Controls	Odds Ratio	Fisher P-Value^2^
**Type**	**Variable**						
**Billed or Problem List Diagnosis**	**F32_Depressive episode**	32	11	(0.021–0.043)	(0.005–0.02)	2.632	**0.005**
	**F41_Other anxiety disorders**	30	10	(0.02–0.041)	(0.004–0.018)	2.714	**0.006**
	**N95_Billed Menopause or perimenopause**	11	1	(0.005–0.018)	(0.0–0.003)	9.952	**0.007**
	**Z72_Problems_related_to_lifestyle**	13	3	(0.006–0.02)	(0.0–0.007)	3.921	**0.024**
	**Anemia dx or problem**	16	5	(0.008–0.024)	(0.001–0.01)	2.895	**0.045**
	**Depression dx or problem**	49	20	(0.036–0.063)	(0.013–0.032)	2.217	**0.003**
	**Sleep dx or problem**	36	15	(0.025–0.048)	(0.008–0.025)	2.171	**0.011**
**Demographics**	**African American**	358	361	(0.333–0.393)	(0.372–0.436)	0.897	0.220
	**Asian**	7	8	(0.002–0.012)	(0.003–0.015)	0.792	0.797
	**Divorced or Sep**	169	130	(0.148–0.195)	(0.122–0.169)	1.176	0.212
	**Married**	476	472	(0.451–0.513)	(0.496–0.561)	0.912	0.264
	**Single**	240	189	(0.216–0.27)	(0.185–0.238)	1.149	0.199
	**White-Caucasian**	599	508	(0.576–0.637)	(0.536–0.601)	1.067	0.404
	**age_60–65**	219	189	(0.196–0.248)	(0.185–0.238)	1.048	0.702
	**age_65–70**	227	227	(0.204–0.256)	(0.226–0.283)	0.905	0.347
	**age_over_70**	110	176	(0.092–0.131)	(0.171–0.223)	0.565	**0.000**
	**age_under_60**	431	301	(0.406–0.468)	(0.306–0.368)	1.296	**0.003**
**Insurance**	**Managed care**	31	16	(0.021–0.042)	(0.009–0.027)	1.753	0.076
	**Medicaid**	17	2	(0.009–0.025)	(0.0–0.005)	7.690	**0.001**
	**Medicare**	179	156	(0.157–0.205)	(0.15–0.2)	1.038	0.767
**Patient History**	**FH breast cancer**	111	122	(0.093–0.132)	(0.114–0.159)	0.823	0.165
	**History of diabetes**	75	68	(0.059–0.093)	(0.059–0.094)	0.998	1.000
	**Neg FH breast cancer**	109	112	(0.091–0.13)	(0.104–0.147)	0.881	0.393

1 Cases are patients, who failed to complete the screening in 180 days, and controls completed the screening within 180 days (excluding same day completion).

2 Significance values below 0.05 are shown in bold.

**Table 4:** Missed screening rates a function of the Rural Continuum Code.

RUCC 2013	Description	Missed Screening	Total Patient Count	Proportion Confidence Int	fishers odds Ratio	fishers P-Value
**2.0**	Metro - Counties in metro areas of 250,000 to …	730	1818	0.379–0.424	1.526	0.0554
**3.0**	Metro - Counties in metro areas of fewer than …	7	12	0.304–0.862	0.698	0.4523
**4.0**	Nonmetro - Urban population of 20,000 or more,…	18	28	0.465–0.82	0.630	0.1403
**6.0**	Nonmetro - Urban population of 2,500 to 19,999…	13	21	0.411–0.827	0.656	0.2543
**7.0**	Nonmetro - Urban population of 2,500 to 19,999…	0	1	0.0–0.0	inf	1.0000

## Data Availability

County and tract level CDC SVI data used in this study is along with age adjusted morality is publicly available and distributed by the CDC^4,6^. The county RUCC codes are publicly available from the USDA^13^. County mammogram screening uptake is publicly available from the Dartmouth Atlas of Healthcare^5^. De-identified patient level data was retrospectively obtained under MUSC IRB Pro00101494. Research using these data was determined by the MUSC IRB board to not to require written consent because presented “no more than minimal risk and involved no procedures.” However, the given the limited use de-identification, the patient level data is not available for public distribution. All methods were carried out in accordance with relevant guidelines and regulations.

## References

[1] CDCBreastCancer. An Update on Cancer Deaths in the United States. Centers for Disease Control and Prevention. Published February 28, 2022. Accessed April 28, 2022. https://www.cdc.gov/cancer/dcpc/research/update-on-cancer-deaths/index.htm

[2] Gerend MA, Pai M. Social Determinants of Black-White Disparities in Breast Cancer Mortality: A Review. Cancer Epidemiol Biomarkers Prev. 2008;17(11):2913–2923. doi:10.1158/1055-9965.EPI-07-0633 18990731

[3] Heller SL, Rosenkrantz AB, Gao Y, Moy L. County-Level Factors Predicting Low Uptake of Screening Mammography. AJR Am J Roentgenol. 2018;211(3):624–629. doi:10.2214/AJR.18.19541 30016143

[4] CDC SVI Documentation 2018 | Place and Health | ATSDR. Published February 10, 2022. Accessed December 17, 2022. https://www.atsdr.cdc.gov/placeandhealth/svi/documentation/SVI_documentation_2018.html

[5] Bronner K, Eliassen MS, King A, Leggett C, Punjasthitkul S, Skinner J. The Dartmouth Atlas of Health Care: 2018 Data Update.:20. 36264873

[6] CDC WONDER. Accessed July 18, 2022. https://wonder.cdc.gov/

[7] Pearl J. An Introduction to Causal Inference. Int J Biostat. 2010;6(2):7. doi:10.2202/1557-4679.1203 PMC2836213 20305706

[8] Padmanaban H. Comparative Analysis of Naive Bayes and Tree Augmented Naive Bayes Models. Master of Science. San Jose State University; 2014. doi:10.31979/etd.n7jg-e3uh

[9] Aliferis CF, Statnikov A, Tsamardinos I, Mani S, Koutsoukos XD. Local Causal and Markov Blanket Induction for Causal Discovery and Feature Selection for Classification Part I: Algorithms and Empirical Evaluation. J Mach Learn Res. 2010;11(7):171–234.

[10] Statnikov A, Lytkin NI, Lemeire J, Aliferis CF. Algorithms for Discovery of Multiple Markov Boundaries. PMC4184048 25285052

[11] Li X, Gao X, Wang C. A Novel BN Learning Algorithm Based on Block Learning Strategy. Sensors. 2020;20(21):E6357. doi:10.3390/s20216357 PMC7664634 33171803

[12] Winzar H. The Ecological Fallacy: How to Spot One and Tips on how to Use One to Your Advantage. Australas Mark J. 2015;23(1):86–92. doi:10.1016/j.ausmj.2014.12.002

[13] USDA ERS - Documentation. Accessed March 29, 2023. https://www.ers.usda.gov/data-products/rural-urban-continuum-codes/documentation/

